# Inhibition of tumour growth by marimastat in a human xenograft model of gastric cancer: relationship with levels of circulating CEA

**DOI:** 10.1038/sj.bjc.6690645

**Published:** 1999-09

**Authors:** S A Watson, T M Morris, H M Collins, L J Bawden, K Hawkins, E A Bone

**Affiliations:** Cancer Studies Unit Department of Surgery, Queen's Medical Centre, Nottingham, UK; British Biotech Pharmaceuticals Limited, Watlington Road Cowley, Oxford, OX4 5LY, UK

**Keywords:** matrix metalloproteinases, marimastat, gastric carcinoma, tumour antigens

## Abstract

Inhibition of matrix metalloproteinases (MMPs) is an attractive approach to adjuvant therapy in the treatment of cancer. Marimastat is the first orally administered, synthetic MMP inhibitor to be evaluated, in this capacity, in the clinic. Measurement of the rate of change of circulating tumour antigens was used for evaluating biological activity and defining optimum dosage in the early clinical trials of marimastat. Although tumour antigen levels have been used in the clinical management of cancer for many years, they have not been validated as markers of disease progression. In order to investigate the relationship between the effects of marimastat on tumour growth and circulating tumour antigen levels, mice bearing the human gastric tumour, MGLVA1, were treated with marimastat. The MMP inhibitor exerted a significant therapeutic effect, reducing tumour growth rate by 48% (*P* = 0.0005), and increasing median survival from 19 to 30 days (*P* = 0.0001). In addition, carcinoembryonic antigen (CEA) levels were measured in serum samples from animals sacrificed at regular intervals, and correlated with excised tumour weight. It was shown that the natural log of the CEA concentration was linearly related to the natural log of the tumour weight and that treatment was not a significant factor in this relationship (*P* = 0.7). In conclusion, circulating CEA levels were not directly affected by marimastat, but did reflect tumour size. These results support the use of cancer antigens as markers of biological activity in early phase trials of non-cytotoxic anticancer agents. © 1999 Cancer Research Campaign


*British Journal of Cancer* (1999) **81**(1), 19–23
© 1999 Cancer Research Campaign
Article no. bjoc.1999.0645

### Inhibition of tumour growth by marimastat in a human xenograft model of gastric cancer: relationship with levels of circulating CEA

**SA Watson[1], TM Morris[1], HM Collins[1], LJ Bawden[2], K Hawkins[2] and EA Bone[2]**

[1]Cancer Studies Unit, Department of Surgery, Queen's Medical Centre, Nottingham, UK; [2]British Biotech Pharmaceuticals Limited, Watlington Road, Cowley, Oxford OX4 5LY, UK

**Summary** Inhibition of matrix metalloproteinases (MMPs) is an attractive approach to adjuvant therapy in the treatment of cancer. Marimastat is the first orally administered, synthetic MMP inhibitor to be evaluated, in this capacity, in the clinic. Measurement of the rate of change of circulating tumour antigens was used for evaluating biological activity and defining optimum dosage in the early clinical trials of marimastat. Although tumour antigen levels have been used in the clinical management of cancer for many years, they have not been validated as markers of disease progression. In order to investigate the relationship between the effects of marimastat on tumour growth and circulating tumour antigen levels, mice bearing the human gastric tumour, MGLVA1, were treated with marimastat. The MMP inhibitor exerted a significant therapeutic effect, reducing tumour growth rate by 48% ($P$ = 0.0005), and increasing median survival from 19 to 30 days ($P$ = 0.0001). In addition, carcinoembryonic antigen (CEA) levels were measured in serum samples from animals sacrificed at regular intervals, and correlated with excised tumour weight. It was shown that the natural log of the CEA concentration was linearly related to the natural log of the tumour weight and that treatment was not a significant factor in this relationship ($P$ = 0.7). In conclusion, circulating CEA levels were not directly affected by marimastat, but did reflect tumour size. These results support the use of cancer antigens as markers of biological activity in early phase trials of non-cytotoxic anticancer agents.

**Keywords:** matrix metalloproteinases; marimastat; gastric carcinoma; tumour antigens

The matrix metalloproteinases (MMPs) are a family of zinc ion ($Zn^{2+}$)-dependent enzymes, which, collectively, are able to degrade all the protein components of the extracellular matrix (ECM) (Matrisian, 1992; Kleiner and Stetler-Stevenson, 1993). The activity of these enzymes is normally tightly regulated in order to control their destructive potential (Cottam and Rees, 1993). In recent years, however, evidence has accumulated that aberrant constitutive expression and activation of MMPs may be important in tumour progression and metastasis (Liotta and Stetler-Stevenson, 1990; Stetler-Stevenson et al, 1996). The breakdown of the ECM surrounding tumours by the action of MMPs would permit local invasion, and the loss of local tissue architecture may promote angiogenesis of the developing tumour. In addition, dissolution of the basement membrane facilitates intravasation and extravasation of tumour cells, essential steps in metastatic dissemination. The hypothesis that MMPs are intimately involved in tumour progression has been supported by studies with various small molecular weight inhibitors of these enzymes, which have been shown to inhibit tumour growth, metastasis and angiogenesis in a number of animal models (Davies et al, 1993; Wang et al, 1994; Watson et al, 1995; Anderson et al, 1996; Eccles et al, 1996; Giavazzi et al, 1998). Several MMP inhibitors have now reached the stage of clinical evaluation. The current study was prompted by the use of cancer antigens in the early trials of one of the first of these inhibitors, marimastat.

*Received 28 August 1998*
*Revised 25 February 1999*
*Accepted 18 March 1999*

*Correspondence to:* EA Bone

Since MMP inhibitors are not cytotoxic, it is predicted that marimastat may cause a reduction in the rate of tumour growth rather than inducing the tumour shrinkage which occurs with conventional, cytotoxic chemotherapeutics. For this reason it was considered likely that standard determinants of tumour response to therapy, namely radiological and clinical measurements, may not detect the clinical activity of marimastat.

An alternative approach, the measurement of changes in circulating levels of serum cancer antigens, was, therefore, used to evaluate the activity of marimastat in early studies. Tumour antigen levels have been used for many years in the clinical management of cancer (Goldenberg et al, 1981). They have proved of value in detecting recurrent disease (NIH Consensus Statement, 1981; Hida et al, 1996), monitoring the effects of chemotherapy and predicting prolonged survival (Hine and Dykes, 1984; Allen-Mersh et al, 1987; Ward et al, 1993). A combined analysis of marimastat clinical trials, in patients with carcinoma of the pancreas, ovary, colon and prostate, has demonstrated a dose-dependent reduction in the rate of rise of cancer antigens (Nemunaitis et al, 1998). These modifications in antigen levels are presumed to reflect changes in the progression of the disease. However, the rate of rise of tumour antigens is an unvalidated measure of disease progression and there has been some controversy over the approach taken in the trials of marimastat (Gore et al, 1996; Millar et al, 1996). It is possible that a decrease in circulating antigen levels may be a direct effect of the inhibitor on the processing of cell surface associated antigens by MMPs or the suppression of cancer antigen production, rather than, or in addition to, inhibition of the progression of the disease.

The purpose of the current study was to investigate the relationship between inhibition of tumour growth by marimastat and changes in circulating carcinoembryonic antigen (CEA) levels.

### MATERIALS AND METHODS

### Cell culture

The effect of marimastat on CEA shedding in vitro was investigated using the human colon adenocarcinoma cell line, LS174T. The cells were seeded at $3 \times 10^5$ per well and incubated at 37°C in a humidified atmosphere of 5% carbon dioxide for 48 h, reaching 80–100% confluence. The medium was removed and the cells washed once with fresh medium. An aliquot of medium containing the appropriate concentration of marimastat was added to the cells, and the incubation was continued for a further 28 h. The supernatants were removed, placed in siliconized tubes, spun and decanted into fresh tubes. Samples were frozen at – 20°C prior to assay.

### Animal handling

Male and female 6 to 8-week-old MF1 *nu/nu* nude mice (Cancer Studies Unit, University of Nottingham) were used in all experiments. The animals were maintained in sterile isolation at 26°C and received food and water ad libitum. All experimentation was performed according to UKCCCR guidelines.

### Tumour

The human gastric cell line MGLVA1 (Watson et al, 1991) is an ascites variant of the gastric carcinoma line, MKN45G. The latter, a gastrin-producing line, is a clonal variant of MKN45, established in vivo in hypergastrinaemic nude mice (Watson et al, 1990). The parental line, MKN45, was derived from a 62-year-old woman with gastric cancer (Hojo, 1977). MGLVA1 was established by growing MKN45G in the peritoneal cavity and passaging the ascitic cells (Watson et al, 1991). This resulted in a more aggressive cell line which reproducibly generated ascites as a monitorable condition during tumour development, rather than just at the end-stage due to high tumour burden. The parental line MKN45 has been used as a CEA-secreting xenograft model (Pimm et al, 1992) and MKN45G has been shown to retain CEA expression (Watson et al, 1991). In the current study we have shown that the ascites variant, MGLVA1, not only expresses CEA, but also that it is shed from the tumour and can be detected in host serum.

MGLVA1 was maintained by serial passage in MF1 nude mice. For experimental purposes, the tumour was removed from donor animals at sacrifice, dissected from the capsule, minced finely into 3-mm$^3$ pieces and pooled. Tumour pieces were implanted subcutaneously into the left flank of an equal number of male and female nude mice under anaesthesia (Hypnorm/Hypnoval, Janssen). Cross-sectional tumour measurements were taken twice weekly from day 5 using callipers, and animals were weighed weekly throughout the study.

In the initial study to examine the effects of marimastat on tumour growth, mice were terminated once their tumour burden was greater than 250-mm$^2$, when their clinical condition became affected.

In the second study, which determined the effect of marimastat on circulating CEA levels, four male and four female animals from both the vehicle- and marimastat-treated groups were sacrificed on days 4, 7, 11, 14, 17 and 21. In addition, marimastat-treated

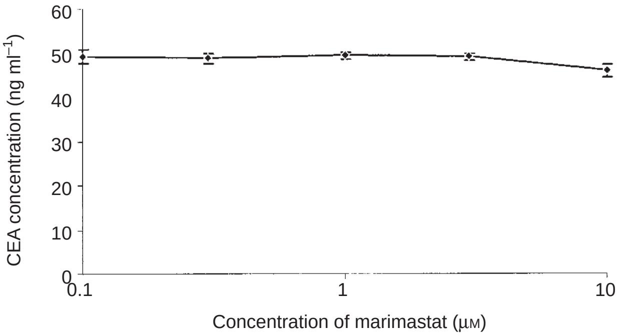

**Figure 1** The effect of marimastat on shedding of cancer antigens from cultured carcinoma cells in vitro animals were sacrificed on days 24, 27 and 30. At all early time points mice were sacrificed randomly. At later time points (day 14 and thereafter) mice bearing tumours exceeding a cross-sectional area of 250-mm$^2$ were selected for termination. At termination, mice were bled by cardiac puncture, the serum collected and frozen at–20°C for subsequent CEA measurement. Tumours were excised and weighed.

### Treatment

Vehicle or marimastat (BB-2516; 2S,N$^1$-dihydroxy-N$^4$-{2,2-dimethyl-1S-[(methylamino)carbonyl]propyl}-3R-isobutylbutan) was administered at 0.5 μl h$^{-1}$ by osmotic mini-pumps (Alzet model 2002, Charles River, Kent, UK) implanted subcutaneously on the right flank at the time of tumour grafting. Pumps were replaced every 14th day. Marimastat was formulated at 15 mg ml$^{-1}$ in 50% dimethyl sulphoxide–water. The dose delivered equates to approximately 6.5 mg kg$^{-1}$ day$^{-1}$.

### CEA measurement

CEA levels in the sera from mice and from cell culture supernatants were measured using the automated IMx® Microparticle Enzyme assay from Abbott Laboratories, according to the manufacturer's recommended protocol. In brief, the sample was incubated with anti-CEA-coated microparticles, to which the antigen binds. An aliquot of the incubation mixture was transferred and bound irreversibly to a glass fibre matrix. Unbound material was removed by washing and an anti-CEA–alkaline phosphatase conjugate was applied to the filter. The conjugate bound to the antibody–antigen complex, and excess conjugate was removed. The substrate, 4-methyllumbelliferyl phosphate was added to the matrix and the fluorescent product measured. Samples were assayed neat, and at a 1 in 5 dilution.

### Statistical analysis

In the first study one mouse was excluded from all analyses as the tumour did not establish. Day 15 was the last day when all mice were left alive. The two-sample *t*-test was used to test for differences between the treatment groups (marimastat or vehicle) in cross-sectional area at day 15.

The time from tumour implant to sacrifice was presented using Kaplan–Meier estimates and the treatment groups were compared using the log-rank test. One mouse was censored as it was sacrificed for reasons other than the cross-sectional area of the tumour.

 © 1999 Cancer Research Campaign

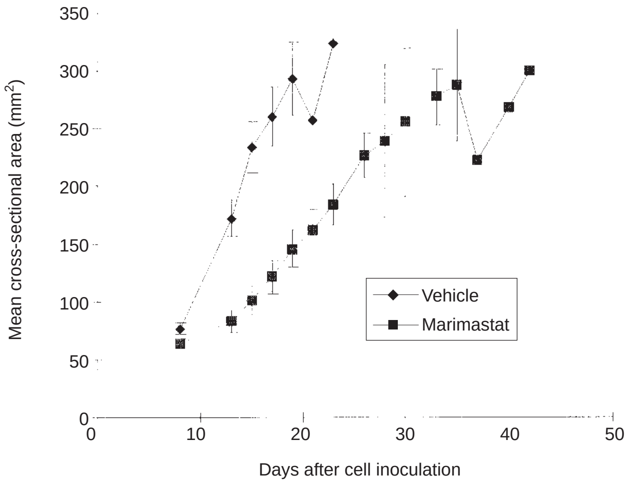

**Figure 2** The effect of marimastat on the increase in cross-sectional area of the human gastric carcinoma, MGLVA1, grown subcutaneously in nude mice

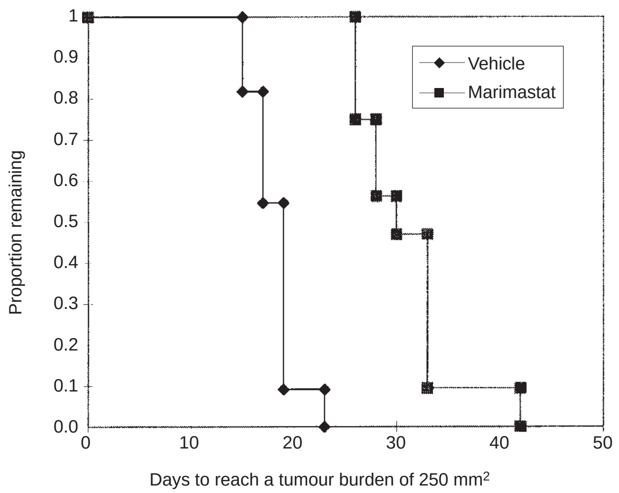

**Figure 3** The time to reach the experimental endpoint in mice bearing the MGLVA1 gastric xenograft

At sacrifice the tumours were excised and weighed. The rate of tumour growth was calculated by dividing the tumour weight by sacrifice time. The treatment groups were compared using the Mann–Whitney *U*-test as the assumptions for the *t*-test were not valid.

The relationship between the CEA levels, tumour weight and treatment (marimastat or vehicle) was investigated using linear regression. The natural log of the CEA levels was used due to the skewness and heteroscedasticity of the data. The natural log of the tumour weight was also used in order to satisfy the assumptions for a linear regression analysis.

### RESULTS

### Effect of marimastat on shedding of CEA from cultured cells

The effect of marimastat on the release of CEA into the culture supernatant of cells was studied using the human colon adenocarcinoma cell line, LS 174T. This line is more amenable to culturing in vitro than the MGLVA1 line which was used in the in vivo models. Concentrations of marimastat, orders of magnitude higher than those achieved in vivo, failed to inhibit the shedding of CEA into the culture supernatant (Figure 1).

### Measurement of circulating CEA

Previous studies have demonstrated that MGLVA1 and its parental line express membrane-bound CEA (Watson et al, 1991). A preliminary in vivo study showed that in blood from nude mice bearing MGLAV1 tumours, CEA was readily detectable; in control animals with mean tumour weight of 2.2 g (s.e.m. = 0.191), the mean concentration of circulating antigen was 548 ng $ml^{-1}$ (s.e.m. = 70.5).

### Effect of marimastat on tumour growth

Treatment of nude mice bearing MGLVA1 xenografts with marimastat significantly inhibited growth of the tumour as measured by cross-sectional area at day 15 (mean cross-sectional area 104.66 $mm^2$ compared with 233.75 $mm^2$ for the vehicle group; $P < 0.0001$, *t*-test). From day 15 onwards animals were sacrificed as their tumour size reached > 250 $mm^2$. Figure 2 shows the mean cross-sectional area over time for the vehicle and marimastat groups. A Kaplan–Meier curve was derived of time from tumour implantation to sacrifice (Figure 3). Marimastat exerted a significant effect ($P = 0.0001$, log-rank test), increasing median time to reach a tumour burden of 250 $mm^2$ from 19 days to 30 days. Marimastat significantly reduced tumour growth rate compared to vehicle-treated animals (0.0572 ± 0.00635 g $day^{-1}$ compared to 0.1098 ± 0.099 g $day^{-1}$; $P = 0.0005$, Mann–Whitney *U*-test).

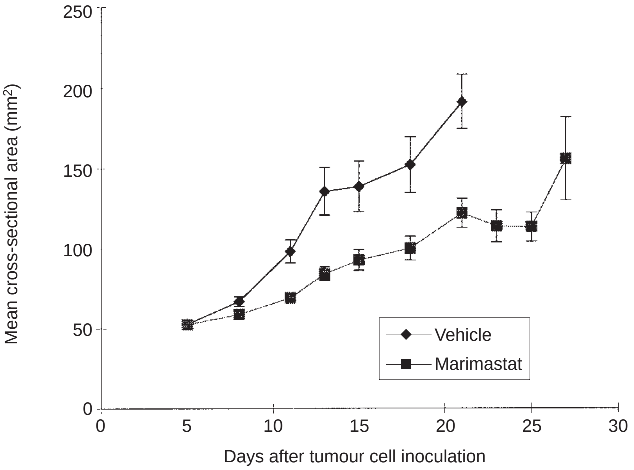

**Figure 4** Confirmation of the inhibitory effect of marimastat on the growth of the gastric carcinoma xenograft

Treatment with marimastat had no effect on animal weight gain over the course of the study compared to vehicle-treated, tumour-bearing controls (data not shown).

The ability of marimastat to inhibit the increase in cross-sectional area of the tumour was confirmed in the second study (Figure 4), which was primarily designed to measure CEA levels

*© 1999 Cancer Research Campaign*

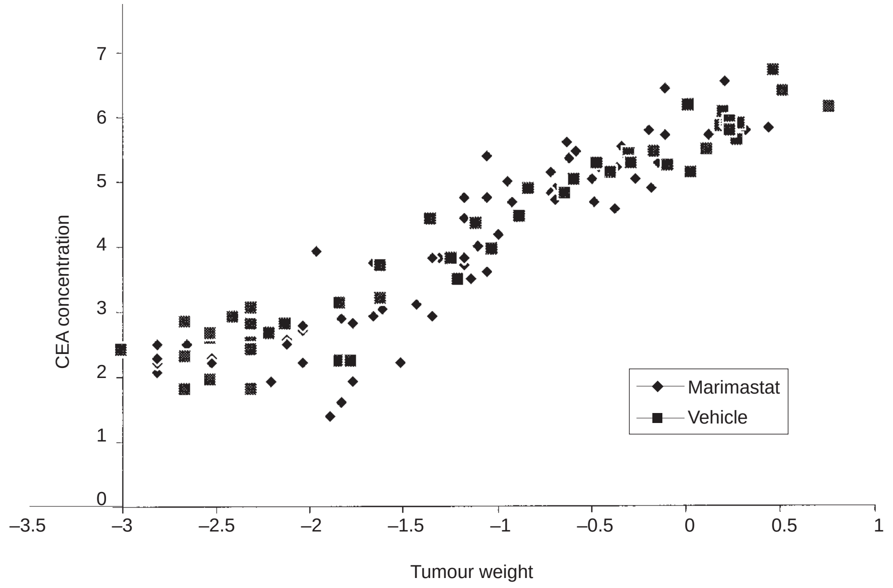

**Figure 5** Relationship between treatment, log of tumour weight and log of circulating carcinoembryonic antigen levels throughout the course of tumour growth. No statistical analysis was performed on the cross-sectional area measurements from this study as a bias was introduced from day 14, after which animals with large tumours were selected to be sacrificed: this tended to reduce the mean tumour weight in the vehicle-treated group to a greater extent than the marimastat-treated group.

### CEA analysis

CEA levels were measured in serum samples from animals sacrificed at regular intervals throughout the course of the second study, and correlated with the excised tumour weight of the appropriate animal. The tumour weights ranged from 0.05 to 2.17 g and from 0.06 to 1.55 g in the vehicle- and marimastat-treated groups respectively. The CEA concentrations ranged from 6 to 809 ng $ml^{-1}$, and from 4 to 679 ng $ml^{-1}$ in the two groups respectively.

The relationship between the CEA levels, tumour weight and treatment was evaluated by linear regression. In order to satisfy the assumptions for such analysis, the natural logs of both CEA concentration and tumour weight were used. The $\log_n$ of CEA concentration was found to be linearly related to the $\log_n$ of the tumour weight by the following formula:

$$\log_n (\text{CEA}) = 5.58 + 1.34 \times \log_n (\text{weight}).$$

It was found that treatment was not a significant term in the model ($P = 0.7$, F-test) and, therefore, was not related to the $\log_n$ CEA levels. The data depicted in Figure 5 show that treatment with marimastat does not influence the distribution of the data set.

### DISCUSSION

Gastric malignancy has previously been shown to be associated with overexpression of MMPs (D’Errico et al, 1991; McDonnell et al, 1991; Honda et al, 1996). The studies described in this report show that marimastat has a marked and significant inhibitory effect upon the growth of the gastric carcinoma xenograft, MGLVA1, subcutaneously implanted in nude mice; measurement of the cross-sectional area of the tumour, rate of increase of tumour weight and time of sacrifice all show the benefit of treatment with marimastat.

In these studies marimastat was delivered by osmotic minipump. In contrast to the pharmacokinetic profile observed in humans (Nemunaitis et al, 1998), the blood concentration of marimastat attained in rodents following oral administration is markedly lower and of shorter duration (unpublished observations). Therefore, in order to demonstrate significant efficacy in rodent cancer models it has been necessary to deliver marimastat continuously by subcutaneously implanted mini-pumps. The differences in metabolism of marimastat between the species also accounts for the apparently higher dose delivered to the mice (6.5 mg $kg^{-1}$ $day^{-1}$) compared to those being used in phase III clinical trials (5–25 mg b.i.d.). The steady-state blood concentration of marimastat in the mice is similar to the mean trough levels achieved in patients.

This study is the first published demonstration of anti-cancer activity for marimastat, and confirms the belief, substantiated by many studies with the related MMP inhibitor batimastat (Davies et al, 1993; Chirivi et al, 1994; Sledge et al, 1995; Taraboletti et al, 1995; Watson et al, 1995), a non-orally bioavailable drug, that MMPIs have promise as anti-cancer therapies.

Levels of circulating CEA have previously been used as a surrogate marker of the therapeutic effect of marimastat in patients with various carcinomas (Millar and Brown, 1996; Nemunaitis et al, 1998; Primrose et al, 1999). Studies were, therefore, instigated to confirm the validity of such an approach. Initial experiments investigating the effect of marimastat on the release of CEA from cells cultured in vitro showed that, even at a very high concentration, the MMPI was unable to inhibit the appearance of CEA in the culture supernatant. These data, whilst interesting, may not necessarily reflect the mechanism of antigen shedding in vivo, as the culture system does not account for the potential involvement of

© 1999 Cancer Research Campaign host factors mediating the release of the antigen from the cell surface. In vivo studies were, therefore, designed to investigate the effect of tumour size and treatment regime on the concentration of circulating CEA.

Animals bearing MGLVA1 tumours were sacrificed at regular intervals throughout the course of the development of the tumours. Groups of animals treated with marimastat were sacrificed at later time points than untreated animals in order to account for the inhibitory effect of marimastat on tumour growth, thereby ensuring that the distribution of tumour size in the treated and untreated groups were similar. As is clear from the graphical depiction (Figure 5), the correlation between tumour weight and circulating CEA levels was independent of whether the samples derived from an animal that had been treated with marimastat or vehicle. These observations were confirmed by statistical methods, which demonstrated that the natural log of CEA concentration was linearly related to the natural log of the tumour weight, and that treatment was not a significant term in the statistical model. This indicates that marimastat has no direct effect on the metabolism of CEA in vivo, and that antigen levels are directly related to tumour size alone. It is reasonable to conclude, therefore, that circulating tumour antigen levels are a valid indicator of the progression of the disease, at least over the period the tumour is growing exponentially, and justifies their use as surrogate markers of biological activity in early phase clinical trials of non-cytotoxic anti-cancer agents.

### REFERENCES

Allen-Mersh TG, Kemeny N, Niedzwiecki D, Shurgot B and Daly JM (1987) Significance of a fall in serum CEA concentration in patients treated with cytotoxic chemotherapy for disseminated colorectal cancer. *Gut* **28**: 1625–1629

Anderson IC, Shipp MA, Docherty AJP and Teicher BA (1996) Combination therapy including a gelatinase inhibitor and cytotoxic agent reduces local invasion and metastasis of murine Lewis lung carcinoma. *Cancer Res* **56**: 715–718

Chirivi RG, Garolafo A, Crimmin MJ, Bawden LJ, Stoppacciaro A, Brown PD and Giavazzi R (1994) Inhibition of the metastatic spread and growth of B16-BL6 murine melanoma by a synthetic matrix metalloproteinase inhibitor. *Int J Cancer* **58**: 460–464

Cottam DW and Rees RC (1993) Regulation of matrix metalloproteinases: their role in tumour invasion and metastasis. *Int J Oncol* **2**: 861–872

Davies B, Brown PD, East N, Crimmin MJ and Balkwill FR (1993) A synthetic matrix metalloproteinase inhibitor decreases tumor burden and prolongs survival of mice bearing human ovarian carcinoma xenografts. *Cancer Res* **53**: 2087–2091

D'Errico A, Garbisa S, Liotta LA, Castronovo V, Stetler-Stevenson WG and Grigioni WF (1991) Augmentation of type IV collagenase, laminin receptor, and Ki67 proliferation antigen associated with human colon, gastric and breast carcinoma progression. *Modern Pathol* **4**: 239–246

Eccles SA, Box GM, Court WJ, Bone EA, Thomas W and Brown PD (1996) Control of lymphatic and hematogenous metastasis of a rat mammary carcinoma by the matrix metalloproteinase inhibitor batimastat (BB-94). *Cancer Res* **56**: 2815–2822

Giavazzi R, Garofalo A, Ferri A, Lucchini V, Bone EA, Chiari S, Brown PD, Nicoletti MI and Taraboletti G (1998) Batimastat, a synthetic inhibitor of matrix metalloproteinases, potentiates the antitumour activity of cisplatin in ovarian carcinoma xenografts. *Clin Cancer Res* **4**: 985–992

Goldenberg DM, Neville AM, Carter AC, Go VL, Holyoke ED, Isselbacher ED, Schein PS and Schwartz M (1981) CEA (carcinoembryonic antigen): its role as a marker in the management of cancer. *J Cancer Res Clin Oncol* **101**: 239–242

Gore M, A'Hern R, Stankiewicz M and Slevin M (1996) Tumour marker levels during marimastat therapy. *Lancet* **348**: 263

Hida J, Yasutomi M, Shindoh K, Kitaoka M, Fujimoto K, Ieda S, Machidera N, Kubo R, Morikawa E, Inufusa H, Watatani M and Okuno K (1996) Second-look operation for recurrent colorectal cancer based on carcinoembryonic antigen and imaging techniques. *Dis Colon Rectum* **39**: 74–79

Hine KR and Dykes PW (1984) Prospective randomised trial of early cytotoxic therapy for recurrent colorectal carcinoma detected by serum CEA. *Gut* **25**: 682–688

Hojo J (1977) Establishment of cultured cell lines of human stomach cancer origin and their morphological characteristics. *Niigata Igakukai Zasshi* **91**: 737

Honda M, Mori M, Ueo H, Sugimachi K and Akiyoshi T (1996) Matrix metalloproteinase-7 expression in gastric carcinoma. *Gut* **39**: 444–448

Kleiner DE Jr and Stetler-Stevenson WG (1993) Structural biochemistry and activation of matrix metalloproteinases. *Curr Opin Cell Biol* **5**: 891–897

Liotta LA and Stetler-Stevenson WG (1990) Metalloproteinases and cancer invasion. *Sem Cancer Biol* **1**: 99–106

Matrisian LM (1992) The matrix-degrading metalloproteinases. *Bioessays* **14**: 455–463

McDonnell S, Navre M, Coffey RJ and Matrisian LM (1991) Expression and localisation of the matrix metalloproteinase Pump-1 (MMP-7) in human gastric and colon carcinoma. *Molecular Carcinogenesis* **4**: 527–533

Millar A and Brown PD (1996) 360 patient meta-analysis of studies of marimastat, a novel matrix metalloproteinase inhibitor. *Ann Oncol* **7**: 123

Millar A, Brown P, Cornish A and Baillet M (1996) Tumour marker levels during marimastat therapy: reply. *Lancet* **348**: 263–264

Nemunaitis J, Poole C, Primrose J, Rosemurgy A, Malfetano J, Brown P, Berrington A, Cornish A, Lynch K, Rasmussen H, Kerr D, Cox D and Millar A (1998) Combined analysis of studies of the effects of the matrix metalloproteinase inhibitor marimastat on serum tumour markers in advanced cancer: selection of a biologically active and tolerable dose for longer-term studies. *Clin Cancer Res* **4**: 1101–1109

NIH Consensus Statement (1981) Carcinoembryonic antigen: its role as a marker in the management of cancer. *Br Med J* **282**: 373–375

Pimm MV, Robins RA and Baldwin RW (1992) Capture of recombinant ricin A chain by a bispecific anti-RTA: anti-CEA monoclonal antibody pre-targeted to a human gastric carcinoma xenograft in nude mice. *J Cancer Res and Clin Oncol* **118**: 367–370

Primrose JN, Bleiberg H, Daniel F, Van Belle S, Mansi JL, Seymour M, Johnson PW, Neoptolomos JP, Baillet M, Barker K, Berrington A, Brown PD, Millar AW and Lynch KP (1999) Pilot study of oral marimastat in recurrent colorectal cancer: an evaluation of biological activity by measurement of carcinoembryonic antigen. *Br J Cancer* **79**: 509–514

Sledge GW Jr, Qulali M, Goulet R, Bone EA and Fife R (1995) Effect of matrix metalloproteinase inhibitor batimastat on breast cancer regrowth and metastasis in athymic mice. *J Natl Cancer Inst* **87**: 1546–1550

Stetler-Stevenson WG, Hewitt R and Corcoran M (1996) Matrix metalloproteinases and tumour invasion: from correlation and causality to the clinic. *Semin Cancer Biol* **7**: 147–154

Taraboletti G, Garofalo A, Belotti D, Drudis T, Borsotti P, Scanziani E, Brown PD and Giavazzi R (1995) Inhibition of angiogenesis and murine hemangioma growth by batimastat, a synthetic inhibitor of matrix metalloproteinases. *J Natl Cancer Inst* **87**: 293–298

Wang X, Fu X, Brown PD, Crimmin MJ and Hoffman RM (1994) Matrix metalloproteinase inhibitor BB-94 (batimastat) inhibits human colon tumor growth and spread in a patient-like orthotopic model in nude mice. *Cancer Res* **54**: 4726–4728

Ward U, Primrose JN, Finan PJ, Perren TJ, Selby P, Purves DA and Cooper EH (1993) The use of tumour markers CEA, CA-129 and CA-242 in evaluating the response to chemotherapy in patients with advanced colorectal cancer. *Br J Cancer* **67**: 1132–1135

Watson SA, Durrant LG and Morris DL (1990) The effect of the $E_2$ prostaglandin enprostil, and the somatostatin analogue SMS 201 995, in the growth of a human gastric cell line, MKN45G. *Int J Cancer* **45**: 90–94

Watson SA, Durrant LG, Wencyk PM, Watson AL and Morris DL (1991) Intracellular gastrin in human gastrointestinal tumor cells. *J Natl Cancer Inst* **83**: 866–871

Watson SA, Morris TM, Robinson G, Crimmin MJ, Brown PD and Hardcastle JD (1995) Inhibition of organ invasion by the matrix metalloproteinase inhibitor batimastat (BB-94) in two human colon carcinoma metastasis models. *Cancer Res* **55**: 3629–3633

*© 1999 Cancer Research Campaign*
